# Cognitive impairment and associated loss in brain white microstructure in aircrew members exposed to engine oil fumes

**DOI:** 10.1007/s11682-015-9395-3

**Published:** 2015-06-12

**Authors:** Liesbeth Reneman, Sanne B. Schagen, Michel Mulder, Henri J. Mutsaerts, Gerard Hageman, Michiel B. de Ruiter

**Affiliations:** Department of Neuroradiology, G1-222, Academic Medical Center, Meibergdreef 9, 1105 AZ Amsterdam, The Netherlands; Brain Imaging Center at the Academic Medical Center, University of Amsterdam, Amsterdam, The Netherlands; Department of Neurology, Solvent Team, Medical Spectrum Twente, Hospital Enschede, Amsterdam, The Netherlands

**Keywords:** Aircrew, Organophosphates, White matter, Injury, MRI, DTI, Cognition, Aerotoxic syndrome

## Abstract

Cabin air in airplanes can be contaminated with engine oil contaminants. These contaminations may contain organophosphates (OPs) which are known neurotoxins to brain white matter. However, it is currently unknown if brain white matter in aircrew is affected. We investigated whether we could objectify cognitive complaints in aircrew and whether we could find a neurobiological substrate for their complaints. After medical ethical approval from the local institutional review board, informed consent was obtained from 12 aircrew (2 females, on average aged 44.4 years, 8,130 flying hours) with cognitive complaints and 11 well matched control subjects (2 females, 43.4 years, 233 flying hours). Depressive symptoms and self-reported cognitive symptoms were assessed, in addition to a neuropsychological test battery. State of the art Magnetic Resonance Imaging (MRI) techniques were administered that assess structural and functional changes, with a focus on white matter integrity. In aircrew we found significantly more self-reported cognitive complaints and depressive symptoms, and a higher number of tests scored in the impaired range compared to the control group. We observed small clusters in the brain in which white matter microstructure was affected. Also, we observed higher cerebral perfusion values in the left occipital cortex, and reduced brain activation on a functional MRI executive function task. The extent of cognitive impairment was strongly associated with white matter integrity, but extent of estimated number of flight hours was not associated with cognitive impairment nor with reductions in white matter microstructure. Defects in brain white matter microstructure and cerebral perfusion are potential neurobiological substrates for cognitive impairments and mood deficits reported in aircrew.

## Introduction

Commercial airliners routinely pump, or “bleed,” compressed air to the cabin that is extracted from aircraft engines or their auxiliary power unit. Nearly all airliners use this “bleed-air” ventilation system. This unfiltered air may sometimes be contaminated with hydraulic fluids, synthetic jet engine oils and/or the compounds released when these fluids and/or oils are heated or pyrolized (for example, carbon monoxide, phosphorus oxides, aldehydes). Medical record review of airline crewmembers who were examined after exposure to contaminated bleed air found acute respiratory and/or central nervous system symptoms among the most commonly reported, including memory impairment, concentration difficulties, gait problems, anxiety, sleep disturbance and depression (Hale and Al-Seffar 2009), sometimes referred to as ‘aerotoxic syndrome’.

Various validated air sampling cases have been conducted on single flights, and low levels of contamination by organophosphates (OP) were observed, including tricresyl phosphate (TCP). However, measurements during leakage of turbine oil into the cabin air (“engine oil fume”) revealed substantially higher TCP contamination (Solbu et al. 2011). For more than a century it is known that OPs such as TCP, widely used as pesticides and developed as chemical warfare nerve agents, are capable to induce brain white matter injury in test animals and humans. OPs are potent inhibitors of the enzyme acetylcholinesterase in the central nervous system (CNS). This causes accumulation of acetylcholine at synapses, cholinergic overstimulation, and eventually degeneration of axons with secondary degeneration of myelin in the peripheral and CNS (Chen 2012).

It is now widely assumed that the potential contamination of aircraft cabin air by engine oil fumes poses a serious aviation safety concern for both aircrew and passengers, mainly because of its detrimental effects on white matter. The past few years this topic has received quite extensive attention in the lay press, following the deaths of two British Airways pilots in January 2013. However, other than incidental reports, medical records or indirect studies using cognitive function tests, the validity of the assumption that engine oil fumes affect the CNS has not been investigated properly.

The purpose of the present explorative study was to determine whether we could objectify cognitive complaints in aircrew using an extensive neuropsychological test battery. And if so, whether we could find a neurobiological substrate for their complaints, using state of the art Magnetic Resonance Imaging (MRI) neuroimaging techniques that assess structural and functional changes, with a focus on white matter integrity, as TCPs are well known to affect axonal integrity, as discussed above (Chen 2012). We used diffusion tensor imaging (DTI) to provide us with information on axonal integrity by measuring the diffusional motion of water molecules in biological tissues using fractional anisotropy (FA). FA is thought to reflect fiber density, axonal diameter, and myelination in CNS white matter (Basser et al. 1994; Le et al. 1992). Although TCPs have been shown to primarily affect the peripheral axons, no studies have yet investigated axonal integrity of CNS, other than studies documenting neurocognitive impairment in aircrew. Only one previous study investigated potential neurobiological substrates of TCPs on the CNS (Heuser et al. 2005) on cerebral blood flow. However, as TCPs have been shown to affect CNS white matter integrity in pre-clinical studies and post-mortem material, FA was our primary outcome measure, and we expected to find a reduction in FA in aircrew members when compared to our control subjects, if indeed TCPs affect CNS white matter integrity. The following secondary outcome measures were selected based on the mode of action of TCPs. Because TCPs and other OPs are potent inhibitors of the enzyme aceylcholinesterase, (Chen 2012) we also investigated brain neurometabolism with proton MR spectroscopy (1H-MRS) including choline (Cho)-containing compounds. In Alzheimer’s disease (AD), loss of cholinergic neurons results in increased levels of free Cho on 1H-MRS, and people with higher Cho/Creatine ratios have a higher risk to develop AD (den Heijer et al. 2006). We expected increased levels Cho/Creatine in aircrew in Cho rich areas: the medial frontal cortex In addition, cerebral blood flow (CBF) was assessed non-invasively using arterial spin labeling (ASL), as cholinesterase inhibition augments CBF, possibly through stimulating effects on the intrinsic Cho cerebrovascular innervations (Claassen and Jansen 2006). Based on a previous report using FDG PET in aircrew members (Heuser et al. 2005), we expected reduced CBF in frontal areas, and increased perfusion in occipital areas. Executive function was tested in the MRI scanner using functional MRI (fMRI). Finally, as the volume of the amygdala and hippocampus are well known neurobiological substrates of cognitive performance and mood (amygdala is reduced in depressed subjects, and hippocampus in AD), we measured their volume using high resolution 3D T1 weighted MR imaging.

## Methods and materials

### Participants

Participants consisted of aircrew members (AC group; pilots and flight attendants and 1 platform supervisor) with cognitive complaints, visiting a clinic for occupational neurological diseases with cognitive complaints, in close time relation with flying hours and for which no other apparent explanation was found as determined by a neurologist from the Solvent Team of the Outpatient Clinic People and Work, Coronel Institute of Occupational Health (GH). AC subjects had a normal neurological examination and were aged between 29 and 55 years of age. Exposure to OPs (flying hours) were estimated using a self-reported questionnaire. Control subjects (C group) consisted of healthy volunteers, predominantly racecar drivers who did not professionally fly, matched for gender, age and IQ. We selected racing-car drivers as we needed a well matched group that had extraordinary response capacities similar to those that are demanded from air crew members, particularly pilots. Exclusion criteria were: a history of neuropsychiatric disease, alcohol abuse, diabetes mellitus, liver and kidney insufficiency, endocrine disease, malignancy, contraindications for MRI, or claustrophobia. The current study was approved by the institutional review board of the Academic Medical Center in Amsterdam. Written informed consent was obtained from all participants according to institutional guidelines after complete description of the study to the subjects.

### Self-report measures and neurocognitive tests

Depressive symptoms were assessed with the Center for Epidemiologic Studies Depression scale (CES-D) (Knight et al. 1997). Subjective cognitive symptoms were assessed with the MOS (Stewart and Ware 1992) scale. Fifteen widely used standardized psychometric neuropsychological tests (comprising 25 test indices) were selected based on their sensitivity for measuring the potential neurotoxic effects of engine oil fumes. The Dutch Adult Reading (DART) test was used as an estimate for premobid verbal intelligence (Schmand et al. 1992). The following tests were administered: Memory: The Dutch version of the Rey Auditory Verbal Learning Test (RAVLT total recall, delayed recall and recognition) (van den Burg et al 1985), Visual Reproduction subtest of the Wechsler Memory Scale – Revised (WMS immediate and delayed recall) (Wechsler 1987). Working Memory: WAIS III Letter-number sequencing and digit span (forward and backward) (Wechsler 2000). Attention: Trail Making test A (Tombaugh 2004) Paced Auditory Serial Addition Test (PASAT) (Spreen and Strauss 1998) Stroop Color Word Test (card 1 and 2) (Hammes 1978) Processing Speed and Reaction Speed: WAIS III digit symbol (Dorfman and Hersen 2001) Fepsy Visual Reaction Time Test and Binary Choice Test (Alpherts and Aldenkamp 1994). Reaction times were evaluated separately on the non-dominant and dominant hand. Motor function: Fepsy Tapping Test (separately for the non-dominant and dominant hand) (Alpherts and Aldenkamp 1994). Verbal Functioning: Phonemic Fluency (D,A,T) (van der Elst et al. 2006) and semantic fluency (animals and occupations) (Luteijn and van der Ploeg 1983) Executive functioning: Trail Making Test B, (Tombaugh 2004) Stroop color-word test card 3, Stroop interference (Hammes 1978) and Wisconsin Card Sorting Test (WCST) (Heaton et al. 1993). The Amsterdam Short-term Memory Test (ASMT) was used as a measure for suboptimal performance (Schagen et al. 1997).

### MR imaging

We used diffusion tensor imaging (DTI) to provide us with information on axonal integrity by measuring the diffusional motion of water molecules in biological tissues using fractional anisotropy (FA). FA is thought to reflect fiber density, axonal diameter, and myelination in CNS white matter (Le et al. 1992, Basser et al. 1994). Because TCPs and other OPs are potent inhibitors of the enzyme aceylcholinesterase, (Chen 2012) we also investigated brain neurometabolism with proton MR spectroscopy (1H-MRS) including choline (Cho)-containing compounds. In Alzheimer’s disease (AD), loss of cholinergic neurons results in increased levels of free Cho on 1H-MRS, and people with higher Cho/Creatine ratios have a higher risk to develop AD (den Heijer et al. 2006). In addition, cerebral blood flow (CBF) was assessed non-invasively using arterial spin labeling (ASL), as cholinesterase inhibition augments CBF possibly through stimulating effects on the intrinsic Cho cerebrovascular innervations (Claassen and Jansen 2006). Executive function was tested in the MRI scanner using functional MRI (fMRI).

Imaging data were obtained at the Academic Medical Center in Amsterdam, using a 3.0 T Intera full-body MRI scanner (Philips Medical Systems, Best, The Netherlands) with a phased array SENSE 8-channel receiver head coil. A sagittal MP-RAGE (magnetization prepared rapid gradient echo (T1W, TR/TE = 6.8/3.1 ms, FOV 252 × 270 mm, 170 slices, voxel size 1.0 × 1.1 × 1.2 mm) was acquired for anatomical reference and volume analysis. Volumes were calculated automatically using the FreeSurfer image analysis suite (http://surfer.nmr.mgh.harvard.edu), as previously described (Fischl et al. 2002). Then, a sagittal 3D FLAIR scan (TR/TE/TI 4,800/355/1,650 ms, FOV 250 × 250 mm, 321 slices, voxel size 1.1 × 1.1 × 0.56 mm, slice gap −0.56 mm) was acquired to score white matter abnormalities with the visual rating scale of Fazekas (range 0–3) (Fazekas et al. 1987). All ratings were performed by a neuroradiologist (L. R.) blind to the clinical data. A single voxel 1H-MRS of 12.0 ml was acquired using a fully automated point resolved spectroscopy (PRESS) sequence positioned in the bilateral medial frontal cortex directly superior to the corpus callosum, TE 2,000 ms, TE of 36 ms, NEX = 64. Following, a DTI sequence was assessed along 16 nonlinear and 16 antipodal directions, as we described elsewhere. (de Ruiter et al. 2012). Pseudo-continuous ASL (pCASL) with background suppression was performed to assess CBF. The applied parameters are described elsewhere (Gevers et al. 2011).

For the fMRI study, we used an fMRI-compatible version of the Tower of London (ToL), a task that reliably activates brain regions associated with executive function, in particular bilateral DLPFC and parietal cortex, in line with a previous study conducted by our group (de Ruiter et al. 2011).

#### Statistical analysis

Demographic variables, self-reported measures, cognitive performance data, Fazekas ratings, and MR spectra were analyzed with SPSS 20.0 (SPSS Inc., Chicago, IL). Demographic and clinical data were analyzed by two-tailed independent-samples t-tests and Chi^2^-tests. MR spectra, ASL perfusion data in frontal and occipital lobes and hippocampal and amygdala volume were analyzed with analysis of covariance (ANCOVA), including age as a covariate. Performance on the 15 neuropsychological tests and the *f*MRI paradigm was also analyzed with ANCOVA, including age and estimated IQ as covariates. Each neuropsychological test score was converted into a standard score by use of the mean and standard deviation of the control group. We classified patients and controls as cognitively impaired or not using the following criteria: first we considered a subject as impaired on a test outcome when the subject scored 2 standard deviations below the mean of the reference group on that test. An overall impairment score was calculated for each subject by counting all tests on which the subject was impaired, as well as the mean number of test scored in the impaired range per group. The fifth percentile of scores in the impaired range (failure on 3 or more tests) of the control group was used as a cutoff for overall impaired or not (Schagen et al. 2006). For the fMRI task, separate analyses were run for performance and reaction times. Differences in FA between the two groups were investigated using voxel-based analysis with SPM8 using general linear model (GLM) with age as covariate. To correct for multiple comparisons, group differences were thresholded at *P* < 0.001 with a minimum cluster size of 10 voxels. To examine the association between extent of cognitive impairment (total number of impaired tests per individual), self-reported complaints (score on MOS scale) and white matter integrity (FA values), and to examine the association between estimated flight hours and extent of cognitive impairment (total number of impaired tests per individual) and FA we used two-tailed Pearson Product Moment Correlations Coefficients.

## Results

### Patient characteristics

The AC group and C group did not differ significantly on gender, age and estimated IQ (Table 1). Both groups did differ on potential exposure to OPs, as the AC group was exposed on average during 8,130 flying hours (lifetime), compared to only 233 h in the control group (*p* < 0.001).

**Table 1:** Patient characteristics, self-report measures and neuropsychological performance

	Aircrew (AC)	Controls (C)	*p*-value
	*n* = 12	*n* = 11	
Female (n)	2	2	
Age (SD) [range]	44.4 (7.1) [29–55]	43.4 (7.1) [32–55]	0.73
Exposure (flying hours)	8130 (3708) [200–15360]	233 (215) [100–750]	<0.001*
DART-IQ (SD) [range]	108.0 (9.0) [98–125]	110.5 (10.2) [87–128]	0.53
Cognitive complaints (MOS)#	3.92 (0.50)	5.0 (0.44)	<0.001*
Depressive symptoms (CES-D)	15.4 (8.8)	6.2 (4.8)	0.006*
CES-D above cut-off (≥16)	8 (58 %)	1 (9 %)	0.01∫*
Neurocognitive impairment (number of tests)*	1.75 (1.14)	0.45 (0.69)	0.007*
Neurocognitive impairment (number of participants)	3 (25 %)	0 (0 %)	0.08∫

∫ Chi square

# lower scores indicate more cognitive complaints

### Self-report measures and neuropsychological performance

#### Cognitive complaints

As expected, the AC group had significantly more self-reported cognitive complaints on the MOS scale (Table 1).

#### Depressive symptoms

With respect to depressive symptoms, the AC group had significantly higher total scores on the CES-D than the C group.

#### Neuropsychological performance

The average number of abnormal tests per group differed significantly between both groups: on average 1.7 tests was abnormal in the AC group, compared to 0.5 in the C group (Table 1). Likewise, the percentage of subjects classified as impaired was higher in the AC compared to the C group: 25 % versus 0 %, respectively. However, this difference did not reach statistical significance.

The groups did not differ significantly in their mean scores on individual neurocognitive outcome measures, except for a working memory measure and two reaction time measure and an interference measure two reaction speed measures (Table 2). On these reaction speed measures and the interference measure, the AC group performed poorer than the C group. On the working memory measure, the AC group performed better than the C group.

**Table 2:** Neuropsychological performance

	Aircrew (AC)	Controls (C)	*p*-value
	*n* = 12	*n* = 11	
Memory function (total scores)			
RAVLT immediate	52.0 (10.2)	54.3 (8.0)	0.73
RAVLT delayed	10.1 (3.2)	10.1 (3.3)	0.62
RAVLT recognition	28.2 (3.4)	29.1 (1.2)	0.57
WMS Immediate	37.3 (2.3)	36.7 (2.6)	0.62
WMS Delayed	34.8 (5.7)	35.3 (3.2)	0.79
Working memory (total scores)			
WAIS Digit span forward	11.6 (2.2)	10.0 (1.8)	0.08
WAIS Digit span backward	9.0 (2.1)	6.8 (1.9)	0.01*
WAIS letter-number sequencing	13.0 (2.0)	12.4 (3.1)	0.50
Attention			
Trailmaking A (sec)#	23.2 (5.3)	22.8 (8.1)	0.96
PASAT (number of errors)#	56.8 (25.0)	88.7 (75.2)	0.21
Stroop color (sec)#	49.0 (7.7)	50.1 (7.7)	0.54
Processing speed and reaction speed			
WAIS digit symbol	77.2 (12.7)	78.0 (13.8)	0.98
Visual DH (msec)#	272.7 (21.5)	252.1 (26.2)	0.09
Visual NH (msec)#	298.0 (46.2)	255.4 (22.6)	0.01*
Binary choice task (msec)#	363.1 (44.7)	313.0 (59.2)	0.05
Motor function			
Finger tapping DH (sec)#	66.6 (7.0)	65.7 (4.7)	0.83
Finger tapping NH (sec)#	59.4 (6.1)	62.7 (7.0)	0.15
Verbal functioning			
Phonemic Fluency (sum score)	43.8 (10.5)	46.0 (16.2)	0.80
Semantic Fluency (sum score)	24.5 (5.4)	26.2 (7.0)	0.54
Executive functioning			
Trailmaking B (sec)#	46.2 (14.2)	48.2 (15.3)	0.66
Stroop color-word (sec)#	82.5 (17.0)	78.7 (15.6)	0.79
WCST perseverations (total score)#	15.8 (17.2)	10.0 (10.0)	0.39
WCST categories (total score)	5.4 (1.4)	5.1 (2.0)	0.68
Stroop interference	1.6 (0.3)	1.4 (0.1)	0.07
Suboptimal performance			
ASMT (total score)	88.8 (1.5)	88.5 (1.5)	0.52
ASMT (range)*	(85–90)	(86–90)	

# lower scores indicate better performance

* cut-off score for noncredible performance 84

### Neuroimaging findings

**Fig. 1:**
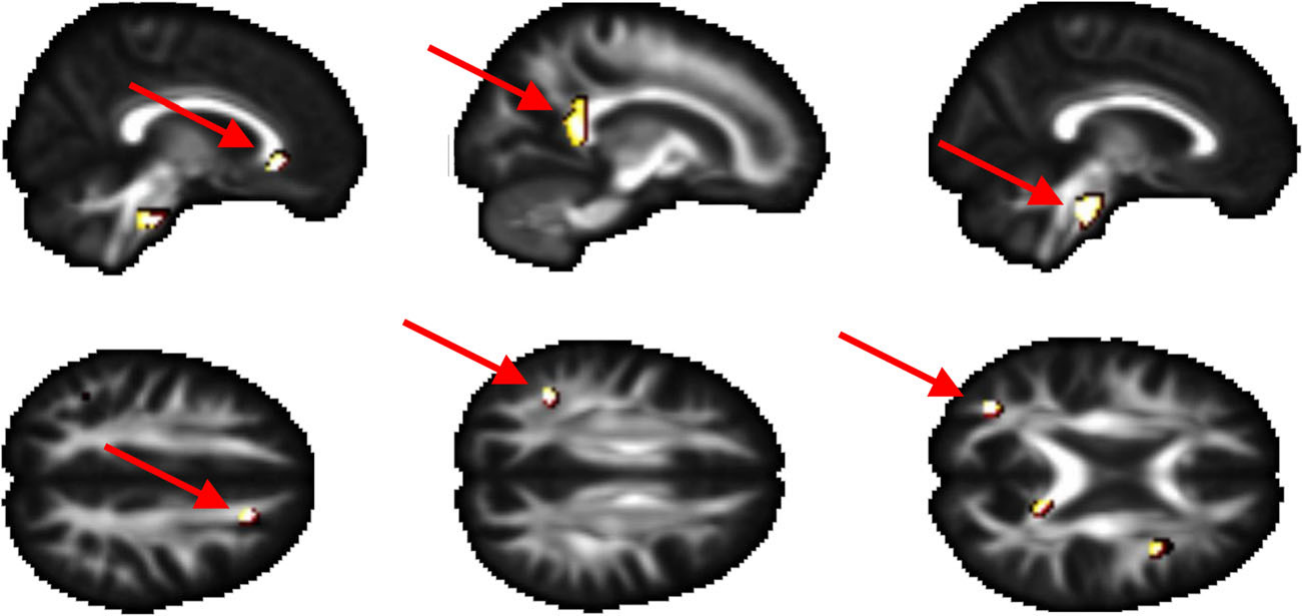
Group differences in microscopic white matter integrity. Statistical parametric map of group differences in microscopic white matter integrity (FA values) between the aircrew (AC) and the controls (C) group. The contrast AC < C is depicted. Significant clusters of lower FA in the AC group are demonstrated in the *upper panel* in the genu of the corpus callosum (*left panel*), the splenium of the corpus callosum (middle panel), and pontine crossing tract (*right panel*). In the *lower panel* clusters are demonstrated in the right anterior corona radiata (*left panel*), left superior longitudinal fasciculus (*middle panel*) and left posterior thalamic radiation (*right panel*). No significant clusters were found for AC > C. Images were thresholded to a cluster significance threshold of *p* < 0.001 and a minimum cluster size of 10 voxels, except for the anterior corona radiata and the superior longitudinal fasciculus (8 and 9 voxels respectively). These clusters are shown because of their direct relation to cognitive functioning. Clusters are shown at *p* < 0.005 to show extent activations

**Fig. 2:**
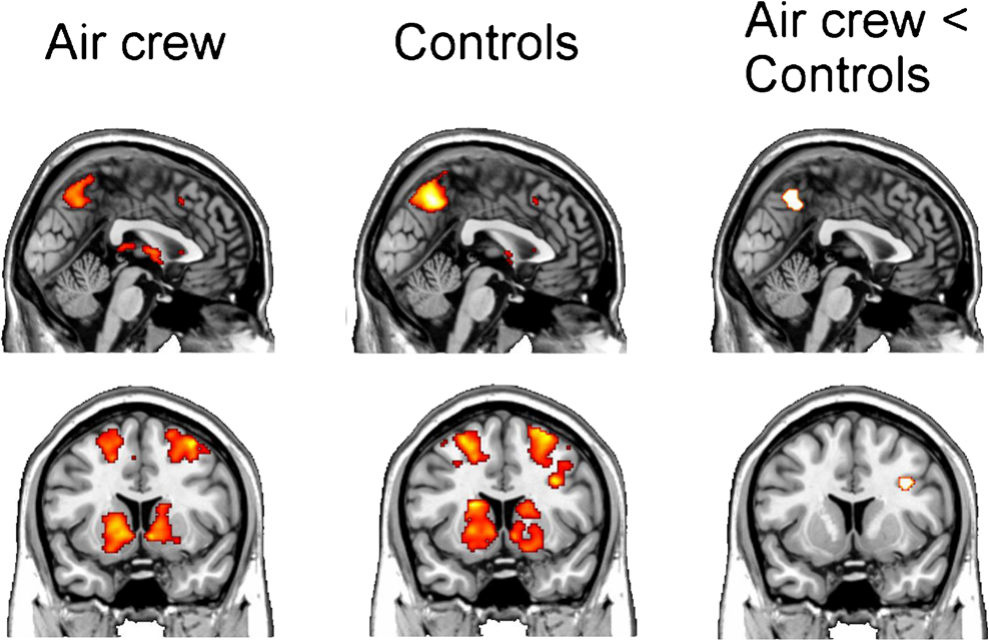
Group differences in Tower of London (ToL) BOLD activations. Statistical parametric map of group differences in Tower of London (ToL). BOLD activations for the Active > Baseline contrast. *Left panel*: task effect for AC. *Middle panel*: task effect for C group. *Right panel*: group interaction AC < C. Upper panel shows hypoactivation in precuneus, lower panel shows hypoactivation for right prefrontal cortex for AC vs. C. Task effects are thresholded at *p* < 0.05 corrected for multiple comparisons . Group interactions were considered significant at *p* < 0.001 with a minimum cluster size of 10 voxels. Group interactions are shown at *p* < 0.005 to show extent activations

Extent of white matter hyperintensities did not differ between both groups (Table 3). As hypothesized, we observed (small but) significantly lower FA values in the AC group compared to the C group in specific white matter regions (Fig. 1). In line with this, we did not find any brain region in which FA was higher than in the C group. No significant differences in brain metabolites nor brain volume were observed between both groups (Table 3). However, we observed a significantly higher CBF in the left occipital cortex (+41.9 %, *p* = 0.04) of the AC group. Also in other brain regions studied, we observed higher CBF in the AC group, but this did not reach statistical significance (Table 3). Finally, no significant differences in performance on the ToL between both groups was observed (93 ± 7 % correct responses in the AC group vs. 89 ± 10 % the C group, *p* = 0.30), also not in reaction time of the responses (11.0 s ± 2.6 versus 11.2 s ± 3.5, respectively, *p* = 0.86). Imaging results showed significant blood-oxygen-level-dependent (BOLD) activation in the (dorsolateral) prefrontal cortex, premotor cortex, dorsal striatum and posterior parietal cortex for the planning vs. baseline contrast (Fig. 2, panels a and b). These effects were found bilateral, and for the AC and C group, demonstrating the robustness of the task in eliciting BOLD activation in relevant brain areas. Significant group differences in BOLD activation where observed in the precuneus and right prefrontal cortex (Fig. 2), in which the AC group displayed significant less brain activation (hypoactivation) when compared to the C group.

**Table 3:** MR imaging outcome: brain neurometabolites, volume and CBF

	Aircrew (AC)	Controls (C)	Delta	*p*-value
	*n* = 12	*n* = 11	AC-C	
Macroscopic inspection white matter				
Fazekas rating 0	10	11	0.16∫	
Fazekas rating 1	2	0		
1H-MRS in left white matter				
Choline/Cr	0.26 (0.02)	0.27 (0.03)	−3.8 %	0.37
NAA + NAAG/Cr	1.30 (0.12)	1.34 (0.14)	−3.0 %	0.38
Brain volume (mL)*				
Total grey matter	683 (47)	683 (73)	0 %	0.70
Total white matter	551 (78)	564 (67)	−2.3 %	0.80
Left hippocampus	4.6 (0.8)	4.8 (0.4)	−4.2 %	0.37
Right hippocampus	4.7 (0.6)	4.8 (0.5)	−2.1 %	0.89
Left amygdala	2.0 (0.3)	1.9 (0.3)	+5.3 %	0.23
Right amygdala	2.1 (0.3)	2.1 (0.3)	0 %	0.29
CBF (mL/100 g tissue/min)				
Overall grey matter	49.4 (19.2)	37.2 (15.6)	+32.8 %	0.06
Overall white matter	16.4 (7.8)	12.4 (6.7)	+32.3 %	0.15
Frontal left cortex	46.6 (18.3)	35.5 (15.7)	+31.3 %	0.07
Frontal right cortex	45.9 (18.3)	34.9 (14.2)	+31.5 %	0.06
Occipital left cortex	45.7 (18.7)	32.2 (15.8)	+41.9 %	0.04*
Occipital right cortex	50.0 (21.4)	36.5 (17.6)	+37.0 %	0.06

∫ Chi square

* corrected for intracranial volume

### Correlation extent of cognitive impairment, self-reported measures and extent of white matter integrity

**Fig. 3:**
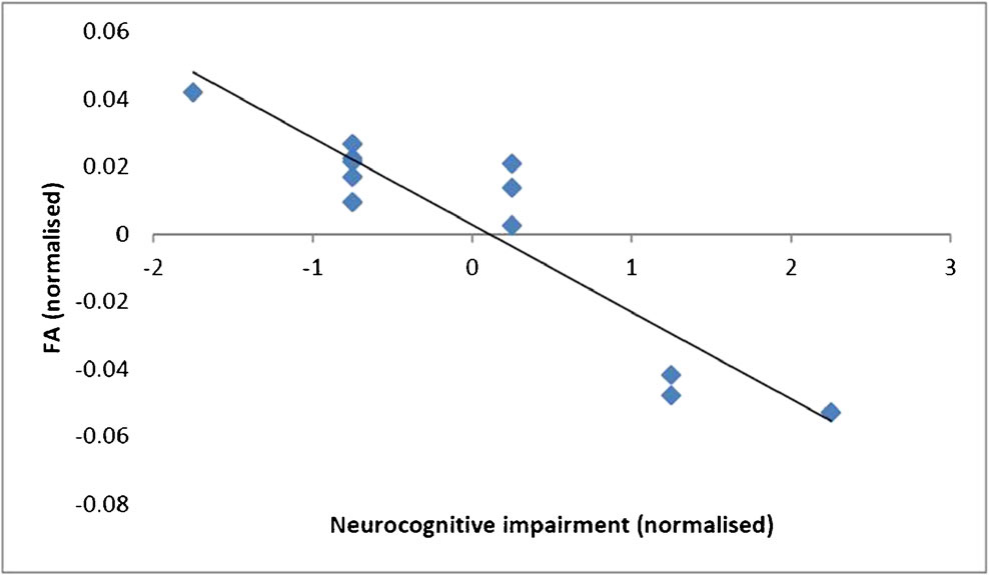
Association between microscopic white matter integrity (FA values) and the extent of cognitive impairment. A significant negative association (*p* < 0.001 one-tailed Pearson Product Moment Correlations Coefficients) between extent of cognitive impairment (total number of impaired tests per individual) and white matter integrity in corona radiata (FA values): the higher the number of abnormal neurocognitive tests of an air crew member, the lower the FA

The extent of cognitive complaints was positively correlated with the extent of abnormal tests (*r* = 0.50, *p* = 0.02): the more cognitive complaints a subject reported, the higher the number of abnormal neuropsychological tests that would be obtained. We observed a similar relationship for depressive symptoms, and also observed that the extent of cognitive complaints predicted the extent of depressive complaints. Even more, the number of neuropsychological tests that the AC group was impaired on was negatively correlated with FA in two white matter regions implicated in cognition. Two significant clusters were identified in the right middle cerebellar peduncle (left panel) and the right posterior corona radiata (illustrated in Fig. 3). The extent of cognitive impairment was associated with estimated number of flight hours, controlled for age at testing (*r* = .12, *p* = .72). Also, the estimated number of flight hours was not associated with reductions in FA, and even showed an inverse trend of reverse association (*r*= .61, *p*= .05)

## Discussion

In aircrew we found significantly more self-reported cognitive complaints and depressive symptoms. Furthermore, aircrew had a significantly higher number of tests scored in the impaired range compared to the control group and also a higher number of aircrew were classified as impaired compared to control subjects, although this difference did not reach statistical significance. Although the white matter macrostructure did not differ significantly between both groups, we observed small brain regions in which brain white matter microstructure was affected (reduced FA values). Also, we observed higher CBF values in the left occipital cortex, as well as hypoactivation in the precuneus and right prefrontal cortex when performing an executive function task. Interestingly, the extent of cognitive impairment correlated with the extent of cognitive complaints and depressive symptoms, as well as white matter integrity. However, the extent of cognitive impairment nor reductions in FA was associated with estimated number of flight hours.

To our knowledge, this is the first study in which neuropsychological functioning of aircrew members was compared to a suitable, matched control group in terms of gender, level of education, estimated IQ, age and profession specific cognitive skills. We found that AC subjects had significantly more cognitive-, and mood complaints than control subjects. Also, the aircrew group scored in the impaired range on a significantly higher number of tests than the control group. It should be noted, however, that the extent of the observed cognitive problems is still quite limited, with only on average 2 tests in the impaired range, and with only a subgroup of aircrew members being classified as cognitively impaired.

We observed reduced BOLD activation in the right prefrontal cortex (PFC) and precuneus in the AC group. Both PFC and precuneus are consistently activated in studies employing the ToL, as well as other executive function tasks (Cabeza and Nyberg 2000). The PFC is mostly linked to executive function, in particular, whereas the precuneus is implicated in visuo-spatial imagery. Thus, our findings suggest reduced neural function during executive function and visuospatial processes in aircrew that is not (yet) apparent on the task performance level. However, one can also conclude that aircrew were more efficient during executive performance.

We observed subtle-, but significant differences in WM integrity using voxel-based whole brain analysis. Reductions in FA most likely reflect microstructural properties and organization of axons (Basser et al. 1994). It has recently been shown that a slight occupational exposure of aircrew to OPs exists (Schindler et al. 2013). OPs induce axonal damage in long nerve axons, such as the spinal cord and peripheral nerves (Chen 2012). The length of the axon is thought to predict vulnerability to the OPs axonal damaging effects. In view of the relatively short axons in the human brain, and the likely limited exposure to OPs in our AC group, the here provided substantial evidence for small but significant damage to axonal microstructure in aircrew members, are in line with these observations. The fact that we observed an association between the extent of cognitive impairment and white matter integrity in aircrew, lends further support to our hypothesis that neurotoxic effects on white matter microstructure may underlie these group differences. Particularly also because it has previously been shown that lower FA values in normal appearing white matter are associated with lower cognitive function (Vernooij et al. 2009). However, we did not observe an association between reductions in FA and estimated number of flight hours.

In line with a previous report (Heuser et al. 2005) we observed a significant increase in brain perfusion in the (left) occipital cortex of the AC group. However, we could not replicate the previously reported *reduction* in frontal brain glucose metabolism assessed using FDG PET. These PET scans, however, were conducted immediately after the subjects had been engaged in a continuous performance task and also did not involve an unexposed control group. With respect to our CBF measurements: it is well known that cholinesterase inhibition augment CBF possibly through stimulating effects on the intrinsic Cho cerebrovascular innervations (Claassen and Jansen 2006). The current increase in occipital brain perfusion in aircrew may therefore be related to previous OP exposure. In line with this, reduced CBF is observed in patients suffering from Alzheimers Disease, which normalizes following treatment with cholinesterase inhibitors (Okonkwo et al. 2014). Finally, no significant differences in brain metabolites nor brain volume were observed between both groups. As discussed also above, the dose to which our AC group was exposed may not have been high enough to induce such structural changes.

In aircrew we found a significantly higher number of cognitive tests scored in the impaired range in aircrew compared to controls, and the extent of cognitive impairment was strongly associated with brain white matter integrity. What are the implications of these findings? First, the present study objectifies cognitive complaints as this group showed a higher number of tests in the impaired range and the extent of this mild impairment correlated with the extent of self-reported measures of cognition and mood. Secondly, we found indicative evidence for a neurobiological substrate for these neuropsychological findings in brain white matter microstructure and cerebral perfusion. It should be kept in mind that our sample size was small and the air crew members consisted of a self-selected group with cognitive and mood complaints. Because we did not observe an association between cognitive impairment and reductions in brain white matter microstructure with estimated number of flight hours, it still needs to be determined in a larger sample size with a longitudinal design if there is a causal link between our observations and exposure to TCPs. We therefore conclude, that in this explorative study, we found preliminary evidence that contamination of aircrew members by engine oil fumes could potentially provide an aviation safety concern that at least warrants further investigations on brain white matter microstructure and cerebral perfusion in larger study populations where alternative explanations like irregular working schedules, and continuous deregulations of the biological clock should also be taken into account to explain the cognitive and mood impairments, in addition to alternative explanations for small clusters of white matter integrity, such as cardiovascular effects, smoking, and exposure to alcohol and drugs (of abuse). A better understanding of these suspected biomarkers may reinforce medical and social recognition and underline the importance of prevention.
